# Harvesting of Prebiotic Fructooligosaccharides by Nonbeneficial Human Gut Bacteria

**DOI:** 10.1128/mSphere.00771-19

**Published:** 2020-01-08

**Authors:** Zhi Wang, Alexandra S. Tauzin, Elisabeth Laville, Pietro Tedesco, Fabien Létisse, Nicolas Terrapon, Pascale Lepercq, Myriam Mercade, Gabrielle Potocki-Veronese

**Affiliations:** a TBI, CNRS, INRA, INSAT, Université de Toulouse, Toulouse, France; b AFMB, UMR 7257 CNRS, Aix-Marseille Université, Marseille, France; c INRA, USC 1408 AFMB, Marseille, France; University of Michigan—Ann Arbor

**Keywords:** *Dorea*, chronic gut diseases, fructooligosaccharides, microbiome, phosphotransferase system

## Abstract

Prebiotic oligosaccharides, such as fructooligosaccharides, are increasingly being used to modulate the composition and activity of the gut microbiota. However, carbohydrate utilization analyses and metagenomic studies recently revealed the ability of deleterious and uncultured human gut bacterial species to metabolize these functional foods. Moreover, because of the difficulties of functionally profiling transmembrane proteins, only a few prebiotic transporters have been biochemically characterized to date, while carbohydrate binding and transport are the first and thus crucial steps in their metabolization. Here, we describe the molecular mechanism of a phosphotransferase system, highlighted as a dietary and pathology biomarker in the human gut microbiome. This transporter is encoded by a metagenomic locus that is highly conserved in several human gut *Firmicutes*, including *Dorea* species. We developed a generic strategy to deeply analyze, *in vitro* and *in cellulo*, the specificity and functionality of recombinant transporters in Escherichia coli, combining carbohydrate utilization locus and host genome engineering and quantification of the binding, transport, and growth rates with analysis of phosphorylated carbohydrates by mass spectrometry. We demonstrated that the *Dorea* fructooligosaccharide transporter is specific for kestose, whether for binding, transport, or phosphorylation. This constitutes the biochemical proof of effective phosphorylation of glycosides with a degree of polymerization of more than 2, extending the known functional diversity of phosphotransferase systems. Based on these new findings, we revisited the classification of these carbohydrate transporters.

**IMPORTANCE** Prebiotics are increasingly used as food supplements, especially in infant formulas, to modify the functioning and composition of the microbiota. However, little is currently known about the mechanisms of prebiotic recognition and transport by gut bacteria, while these steps are crucial in their metabolism. In this study, we established a new strategy to profile the specificity of oligosaccharide transporters, combining microbiomics, genetic locus and strain engineering, and state-of-the art metabolomics. We revisited the transporter classification database and proposed a new way to classify these membrane proteins based on their structural and mechanistic similarities. Based on these developments, we identified and characterized, at the molecular level, a fructooligosaccharide transporting phosphotransferase system, which constitutes a biomarker of diet and gut pathology. The deciphering of this prebiotic metabolization mechanism by a nonbeneficial bacterium highlights the controversial use of prebiotics, especially in the context of chronic gut diseases.

## Abstract (precis)

Prebiotics are increasingly used as food supplements, especially in infant formulas, to modify the functioning and composition of the microbiota. However, little is currently known about the mechanisms of prebiotic recognition and transport by gut bacteria, while these steps are crucial in their metabolism. In this study, we established a new strategy to profile the specificity of oligosaccharide transporters, combining microbiomics, genetic locus and strain engineering, and state-of-the art metabolomics. We revisited the transporter classification database and proposed a new way to classify these membrane proteins based on their structural and mechanistic similarities. Based on these developments, we identified and characterized, at the molecular level, a fructooligosaccharide transporting phosphotransferase system, which constitutes a biomarker of diet and gut pathology. The deciphering of this prebiotic metabolization mechanism by a nonbeneficial bacterium highlights the controversial use of prebiotics, especially in the context of chronic gut diseases.

## INTRODUCTION

Prebiotics are functional foods, defined as “nondigestible compounds that, through their metabolization by microorganisms in the gut, modulate composition and/or activity of the gut microbiota, thus conferring a beneficial physiological effect on the host” (1–3). Inulin and fructooligosaccharides (FOS), comprising β(2→1) fructosyl units linked to sucrose, are universally agreed to be prebiotic fibers. They are naturally present in a variety of plants such as Jerusalem artichoke, wheat, barley, rye, onions, garlic, asparagus, and banana (4, 5). They are increasingly used as food supplements, especially in infant formulas (5, 6), with the global inulin and FOS markets estimated to exceed $5.8 billion by 2024 (7, 8). Numerous studies indicate that inulin and FOS are selectively fermented by human gut bifidobacteria and lactobacilli, inducing beneficial effects for the host, such as facilitating defecation and reducing endotoxemia, insulin resistance, and colon cancer risks (9–12). Notwithstanding this evidence and the commercial interest, some nonbeneficial gut bacterial species (13, 14), such as Dorea longicatena, a species associated with several pathologies such as irritable bowel syndrome (IBS) (15, 16), have been shown to feed on these compounds. Moreover, abdominal pain in IBS is alleviated by limiting the consumption of fermentable oligo-, di-, and monosaccharides and polyols (FODMAPs) such as FOS, resulting in a decrease in *Bacteroides*, *Ruminococcaceae*, *Faecalibacterium*, and *Dorea* species in the gut microbiota composition (17). In addition, the abundance of *Dorea* species increases after fermentation of the human fecal microbiota on FOS (18). Dorea longicatena is stimulated in mouse fecal microbiota after mice were transplanted with a human microbiota and fed FOS (19). Furthermore, we previously demonstrated that many prominent uncultured human gut bacteria, of which the benefits or risks for human health are not known, are well equipped to metabolize prebiotics, including FOS (20). Besides, several pathogenic Escherichia coli strains, including strain BEN2908 that has the ability to invade human epithelial cells (21), can metabolize FOS (13, 22). In any event, regardless of their microbial origins, there has been little investigation of the mechanisms of FOS metabolization at the molecular level.

In bacteria, the complete metabolization of glycosides requires at least a set of carbohydrate-active enzymes (CAZymes; http://www.cazy.org/) (23) and one or more specific transporters (24, 25). This complex catabolic machinery is encoded by genes clustered within the same genomic loci (26, 27), called polysaccharide utilization loci (PULs) for *Bacteroidetes* (28). This name is further extended to Gram-positive bacteria, known as Gram-positive PULs (gpPULs) (29). As with the other glycans, the proposed mechanism of inulin and FOS metabolization requires four steps (30): (i) extracellular hydrolysis of the longest chains, by cell surface-associated inulinases and/or fructosidases, releasing monosaccharides or short oligosaccharides; (ii) internalization of these short compounds by transporters; (iii) final depolymerization of the oligosaccharides by intracellular fructosidases; and (iv) monosaccharide assimilation in the central metabolism. A variety of bacterial inulin-targeting *Bacteroidetes* PULs (31, 32) and gpPULs (33) have been identified in the past few decades, and many inulinases and fructosidases have been biochemically characterized (34). However, only a few transporters have been proven to internalize FOS: the ATP-binding cassette (ABC) transport systems from the probiotic strain Lactobacillus acidophilus NCFM (35) and the oral pathogenic strain Streptococcus pneumoniae TIGR4 (36), the major facilitator superfamily (MFS) transporter from the extraintestinal pathogenic Escherichia coli strain BEN2908 (22), and two phosphotransferase systems (PTSs) from the probiotic strain Lactobacillus plantarum ST-III (37). Nevertheless, the binding and transport specificity of these transporters have still not been determined.

There are, in fact, several technological barriers to the functional characterization of transporters in native bacteria. Despite major advances for some bacterial genera over the last 10 years (38, 39), the difficulties of genetic manipulation, often combined with functional redundancy, render it challenging to characterize transport systems. In the case of uncultured species, it is impossible to inactivate or delete genes. Notably, these species make up more than 70% of the human gut ecosystem (40). In addition, transmembrane proteins cannot be studied as easily as soluble proteins. Consequently, experimental validation of function has taken place for only a few glycoside transporters, while there are probably hundreds of thousands in bacteria, considering that the PUL database lists more than 30,000 *Bacteroidetes* sequences for the SusC/D transporter family alone from 1,154 species (28).

Here, we present a highly generic approach to *in vitro* and *in cellulo* characterization of the binding and transport specificities of membrane-bound proteins based on heterologous expression in E. coli. Our strategy combines PUL engineering, analysis of growth dynamics, characterization of transporter functionality, and quantification of binding and transport rates. This novel strategy allowed us to deeply characterize a FOS PTS transporter issued from a dominant uncultured *Dorea* strain, previously identified by mining the human ileal microbiome for prebiotic metabolization pathways (20). This *Dorea* FOS utilization system is the first prebiotic metabolization pathway to be characterized from a nonbeneficial human gut bacterium.

## RESULTS

### Organization of the *Dorea* FOS utilization locus.

**FIG 1:**
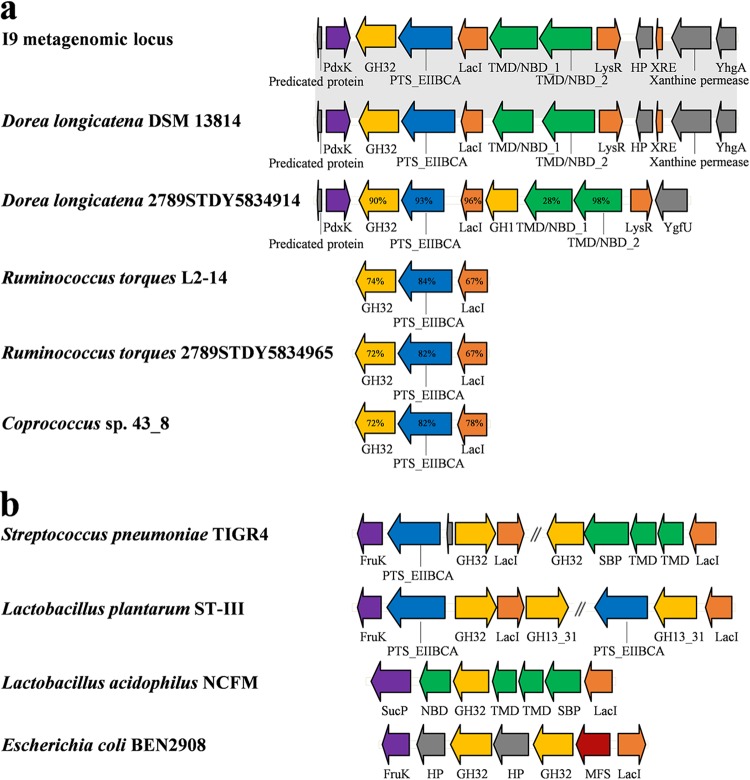
Representations of FOS and sucrose utilization loci. (a) Alignment of the I9 metagenomic FOS utilization locus with similar loci from cultured bacteria. Synteny between the I9 sequence and the genomic locus of *D. longicatena* DSM 13814 is shown by gray bars. Percentages of identity are indicated when lower than 100%. (b) Alignment of the characterized FOS and sucrose utilization loci involving either an ABC, an MFS, or a PTS transport system, a GH32, and a related regulator. Genes predicted as being involved in carbohydrate sensing, transport, and hydrolysis are color coded: glycoside hydrolases (GH) in yellow, PTS in blue, ABC transporter components in green (NBD, nucleotide binding domain; TMD, transmembrane domain; SBP, solute binding protein), MFS in red, LacI regulator and XRE (xenobiotic response element) family transcriptional regulator in orange, and PdxK (pyridoxine kinase), FruK (fructokinase), and SucP (sucrose phosphorylases) in purple. The other genes are indicated in gray: hypothetical protein (HP), xanthine permease, YhgA (putative YhgA-like transposase), and YgfU (putative purine permease).

By means of activity-based screening of the human ileal metagenome, we previously identified a fosmid E. coli clone (clone I9) producing intracellular fructosidase activity responsible for the hydrolysis of a mixture of FOS, containing 1-kestose (GF_2_), nystose (GF_3_), and 1^F^-fructofuranosyl-nystose (GF_4_) (20). After sequencing the I9 metagenomic DNA, two contigs were obtained, I9a and I9b, with sizes of 14,714 and 13,700 bp, respectively. The I9a contig encodes a FOS utilization locus, containing genes annotated as encoding a glycoside hydrolase belonging to the family GH32 of the CAZy classification, a PTS component, a transcriptional regulator of the LacI family, and two ABC transporter proteins (Fig. 1a). No peptide signal was identified in the GH32 sequence, indicating a cytoplasmic location of this enzyme in the native organism. The RPS-BLAST analysis against the NCBI’s conserved domain database (CDD) indicated that both proteins annotated as ABC transporters are multidomain proteins, each with a transmembrane domain (TMD) and a cytosolic nucleotide-binding domain (NBD), which together constitute a complete ABC transporter that nevertheless lacks a solute binding protein (SBP). The same bioinformatic analysis of the PTS transporter sequence of the I9a contig was performed. PTSs are multiple-component carbohydrate uptake systems, allowing the phosphoryl group from phosphoenolpyruvate (PEP) to be successively transferred from enzyme I (EI) to the histidine protein (HPr), then to the domains A and B of enzyme II (EII), and finally to the carbohydrate bound to the EII C domain (41). The I9 PTS sequence analysis revealed that it is a single multidomain protein, composed of the three domains B, C, and A of the PTS EII complex. We thus named it PTS_EIIBCA. The I9 FOS utilization locus lacks genes encoding EI and HPr. As EI and HPr have been proven to be nonspecific and shared by different PTS systems (42, 43), we hypothesized that E. coli EI and HPr could complement the I9 PTS_EIIBCA. Moreover, no transmembrane helix was predicted in the EIIA and EIIB domains, but ten were found in the EIIC domain, suggesting that the EIIC domain is membrane spanning and could potentially bind extracellular substrates.

The I9a nucleic acid sequence presents 99% identity on 93% of its length with a part of the Dorea longicatena DSM 13814 genome (Fig. 1a). In addition, the I9a sequence presents a microsynteny, covering the GH32- to the LacI-encoding genes, with several human gut *Firmicutes* genomes, such as Dorea longicatena, Ruminococcus torques, and *Coprococcus* sp., which all belong to the *Clostridiales* order (Fig. 1a). To date, no FOS utilization locus has been characterized from *Clostridiales*, even though several species in this order have been proven to metabolize FOS *in vitro* (13, 44). In contrast, several other *Firmicutes* loci harboring just one of the PTS or ABC transport systems associated with at least one GH32 and a regulator have been proposed to be involved in the utilization of FOS by *Lactobacillus* spp. using transcriptomic analysis, sometimes combined with gene inactivation (Fig. 1b) (35, 37, 45). In Streptococcus pneumoniae, two GH32-containing loci, the *scr* locus including a PTS transporter and the *sus* locus including an ABC transport system, were shown to be responsible for the utilization of sucrose and FOS, respectively (Fig. 1b) (36). Here, though, we observed both transport systems localized in the same locus. The presence within the same locus of both transport systems suggests a difference in the specificities of the I9 ABC and PTS.

### Abundance of the *Dorea* FOS utilization locus in the human gut microbiome.

**FIG 2:**
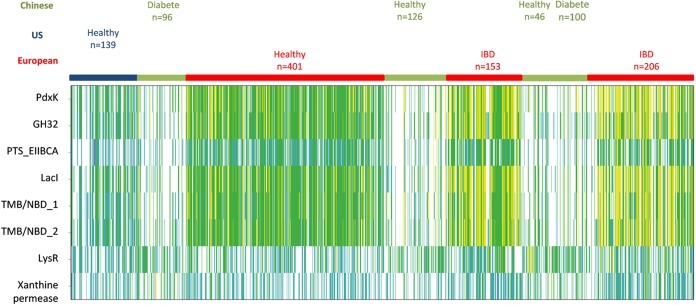
I9 FOS utilization locus abundance and prevalence in the human gut microbiome. Gene abundance in the fecal metagenome is represented by a color scale: white, not detected; blue, turquoise, green, yellow, orange, and red, increasing abundance with a 10-fold change between colors. Based on its annotation, the xanthine permease-encoding gene is not involved in carbohydrate binding, sensing, transport, or modification.

The prevalence and abundance of the genes within the *Dorea* FOS utilization locus in the human gut microbiome were quantified from the abundance table of the human gut metagenomic gene catalogue (Fig. 2) built from fecal samples from 1,267 individuals of various geographic origins (America, Europe, and China), lifestyles, and medical statuses (46).

We found that eight genes of the I9 locus have homologs (with more than 97% sequence identity and 100% coverage) in the microbiome. These genes are highly prevalent, being present in at least 50% of the individuals in the cohort. Interestingly, the *pts_EIIBCA* gene is 4.6 times less abundant than the other genes of the locus, although with a similar prevalence. This result indicates that the structure of the *Dorea* FOS locus has been altered during its evolution, which might be due to (i) the *a posteriori* gene acquisition of the *Dorea pts* gene from an existing locus or (ii) its loss within some bacterial populations over time. Both events could have been driven by horizontal gene transfer, an essential phenomenon for bacterial genome plasticity and metabolic adaptability (47). Nevertheless, when comparing gene abundance and prevalence in the microbiome of individuals of various origins and medical statuses, the *pts* gene presents the same relative profile as the other genes of the FOS utilization locus. The six genes are indeed on average 3 and 6.5 times more prevalent and abundant, respectively, in Westerners (including Americans and Europeans) than in individuals of Chinese origin. This is probably due to the dietary differences between Westerners and Asians. Wheat is a major source of FOS (5), and bread made with wheat is a staple food for Westerners (48, 49), while for Asians, it is rice (50, 51).

In addition, in the European cohort, these genes are similarly prevalent but twice as abundant in the microbiomes of patients suffering from inflammatory bowel diseases (IBDs; including Crohn’s disease and ulcerative colitis) than in those of healthy individuals. This suggests that bacteria possessing this FOS utilization locus may be positively associated with IBD.

### The *Dorea* phosphotransferase system confers FOS utilization ability to E. coli.

First, in order to identify which genes expressed in E. coli could explain the *Dorea* I9 FOS utilization phenotype, the gene expression levels were quantified by reverse transcription-quantitative PCR (RT-qPCR) for the four genes potentially involved in substrate internalization (*pts_EIIBCA*, *tmb/nbd_1*, and *tmb/nbd_2*) and hydrolysis (*gh32*) (see Fig. S1 in the supplemental material). Except for *tmb/nbd_2*, which displayed a relatively low expression, all the genes were expressed at increased levels compared to that of the endogenous E. coli housekeeping gene (*ihfB*). The moderate expression of the ABC transporter-encoding genes suggests that the ability of E. coli to internalize FOS might be mostly due to the expression of the *pts_EIIBCA* gene. In addition, this transcriptomic study highlights the potential of E. coli to properly express *Firmicutes* genes cloned in fosmids. This result is consistent with our previous study performed with a *Bacteroidetes* PUL, demonstrating that a significant proportion of metagenomic genes could be expressed spuriously in E. coli due to the presence within the metagenomic sequence of numerous σ^70^ promoter sequences for E. coli (52).

10.1128/mSphere.00771-19.1 FIG S1 Relative expression levels of genes conferring the FOS metabolization phenotype to the I9 clone versus that of the endogenous E. coli housekeeping gene (*ihfB*). The data represent four technical replicates from three biological replicates. Download FIG S1, TIF file, 2.9 MB. Copyright © 2020 Wang et al. 2020 Wang et al. This content is distributed under the terms of the Creative Commons Attribution 4.0 International license.

**FIG 3:**
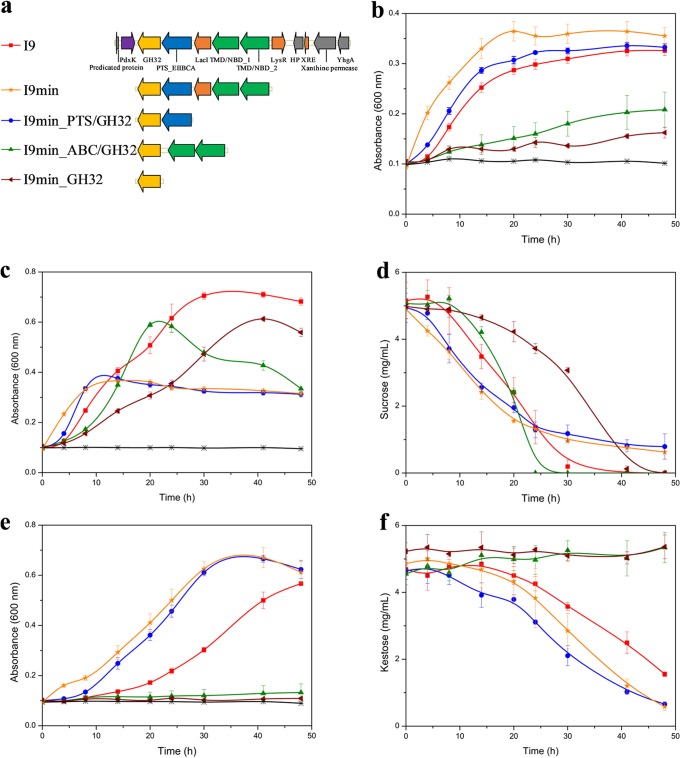
Functional analysis of the I9 FOS utilization locus. (a) Gene organization in the I9 variants. Growth curves of clone I9, its variants, and the control clone EPI on FOS mixture (b), sucrose (c), and kestose (e). HPAEC-PAD analysis of the culture supernatants for growth on sucrose (d) and kestose (f). Color code: clone I9, red; I9min, yellow; I9min_PTS/GH32, blue; I9min_ABC/GH32, green; I9min_GH32, brown; EPI, black. The symbols indicate the sampling time points. The data represent the averages from biological triplicates.

Second, in order to establish the contribution of each gene in the *Dorea* FOS locus in the ability of E. coli to metabolize FOS, different truncated variants of the I9 metagenomic DNA insert were generated (Fig. 3a). The E. coli host transformed with the empty fosmid pCC1FOS, here named EPI, was used as a negative-control strain.

We showed that only the I9 GH32 enzyme was responsible for FOS hydrolysis by testing cytoplasmic cell extracts of the I9 variant harboring only the GH32 (I9min_GH32) on pure sucrose, kestose, nystose, fructosyl-nystose, inulotriose, inulin, and levan. Among these carbohydrates, only sucrose, kestose, nystose, fructosyl-nystose, and inulotriose were hydrolyzed (Fig. S2; see also Table S2 in the supplemental material). Moreover, this enzyme prefers sucrose and FOS up to degree of polymerization 4 (DP4) to longer oligosaccharides, since fructosyl-nystose was not fully hydrolyzed and even present at a lower molar concentration than nystose and kestose (Table S2). In contrast, EPI cytoplasmic extracts displayed no activity on the same substrates, validating the fact that the E. coli EPI100 host strain was not able to hydrolyze FOS. In addition, no secreted activity was detected in I9min_GH32 (data not shown), confirming that the GH32 enzyme is produced exclusively inside the E. coli cells.

10.1128/mSphere.00771-19.2 FIG S2 HPAEC-PAD analysis of the reaction products resulting from FOS (a) and inulotriose (b) hydrolysis by the I9min_GH32 cytoplasmic extracts after 24 h of incubation at 37°C. Download FIG S2, TIF file, 2.8 MB. Copyright © 2020 Wang et al. 2020 Wang et al. This content is distributed under the terms of the Creative Commons Attribution 4.0 International license.

To determine the internalization specificity of the two I9 transporters (the PTS and the ABC transport system), the ability of truncated variants to use FOS as the sole carbon source was assessed (Fig. 3b). First, by constructing an I9-reduced variant consisting of the genes from *gh32* to *tmb/nbd_2* (I9min), we confirmed that only this minimal locus was responsible for the growth on FOS. The I9 and I9min clones in fact displayed similar growth on FOS. The slight difference might result from the high number of recombinant proteins that have to be produced in I9, which slowed down its growth compared to that of I9min. Interestingly, the clone I9min_GH32 grew slowly on the FOS mixture. This result indicates that the EPI100 host strain possesses at least one nonspecific transporter able to internalize at least one of the components of the FOS mixture that is further metabolized by the host after hydrolysis by the intracellular GH32. The I9min_PTS/GH32 variant, harboring the PTS transporter and the GH32, retained the growth ability of I9 on FOS. In contrast, the growth of I9min_ABC/GH32 on FOS was strongly impaired, with a growth rate barely higher than that of I9min_GH32. The modest growth of I9min_ABC/GH32 might be due to the uptake of sucrose from the FOS mixture, but at this stage of the study, we could not exclude that the ABC transporter has a very low ability to transport FOS.

To characterize the transport specificity of each I9 transport system more precisely, for each variant, we monitored the consumption of individual FOS components in the culture medium using high-pH anion-exchange chromatography–pulsed amperometric detection (HPAEC-PAD) (see Fig. S3). First, as proposed above, the host strain EPI100 possesses its own sucrose transporter, since the amount of sucrose slightly decreased over time for I9min_GH32. When growing on the FOS mixture, clones I9, I9min, and I9min_PTS/GH32 preferentially metabolized sucrose (GF) and kestose (GF_2_), while nystose (GF_3_) and 1^F^-fructofuranosyl-nystose (GF_4_) were not used. In contrast, I9min_ABC/GH32 consumed only sucrose. No increase in the oligosaccharide concentration was observed in the culture medium during growth, indicating that the I9 ABC and PTS transporters are both importers, contrary to what was observed with similar analyses for other transport systems, such as those from the major facilitator superfamily (MFS) (52). To confirm the specificity of the transporters either for sucrose or kestose, the uptake of these two oligosaccharides was examined by growth test and HPAEC-PAD analyses (Fig. 3c to e and f). All the variants were able to use sucrose to grow except EPI. However, sucrose appears not be the favorite substrate for the PTS transporter, as the growth rate of I9min_PTS/GH32 on sucrose was lower than for I9. I9min_ABC/GH32 grew more rapidly than I9min_GH32 on sucrose, but these PTS-devoid clones were unable to use kestose. This demonstrates that even if the host strain possesses its own sucrose transporter, the I9 ABC transporter has the ability to transport sucrose. As far as we know, it is the first example of carbohydrate importer of the ABC transporter family which lacks an SBP (53). When the SBP is lacking, such as in ABC transporters catalyzing micronutrient uptake (54), the TMDs determine the specificity of the transporter through substrate-binding sites. Nevertheless, the mechanism explaining how the I9 ABC transporter could import sucrose without SBP remains to be characterized.

10.1128/mSphere.00771-19.3 FIG S3 Analysis of the uptake of each component of the FOS mixture by clone I9 (a) and its variants I9min (b), I9min_PTS/GH32 (c), I9min_ABC/GH32 (d), and I9min_GH32 (e). The different FOS components are color coded: sucrose (GF), blue; kestose (GF_2_), green; nystose (GF_3_), red; and 1^F^-fructofuranosyl-nystose (GF_4_), black. The data represent two biological replicates. Download FIG S3, PDF file, 0.2 MB. Copyright © 2020 Wang et al. 2020 Wang et al. This content is distributed under the terms of the Creative Commons Attribution 4.0 International license.

To deeper analyze the specificity of the PTS and ABC transporters to fructans and fructooligosaccharides, the growth of I9 was also tested on inulin (GFn with β-1,2-linked fructosyl units), levan (GFn with β-2,6-linked fructosyl units), and inulotriose (F3 with β-1,2-linked fructosyl units). No growth was observed with any of these carbohydrates (data not shown). However, we found that the GH32 enzyme was able to hydrolyze inulotriose *in vitro* (see Fig. S2b and Table S2), demonstrating that the metabolization of inulotriose by I9 is blocked at the transport step. From these results, we conclude that (i) the I9 PTS is specific to sucrose and kestose (with a clear preference for kestose) and requires a terminal glucosyl moiety to recognize the oligosaccharide to be internalized and (ii) the I9 ABC transporter is able to transport sucrose only.

### Binding specificity of the *Dorea* PTS transporter.

To determine the transport and binding specificities of the PTS transporter, we had to establish a new methodology. Up until now, the characterization of transporter specificity, performed *in vivo* by growth analysis after knockout (55), the determination of the internalization rate of radiolabeled substrates (56), and/or transcriptional analysis (57), did not allow for separate analyses of the binding, transport, and metabolization specificities. In addition, the substrate of transcription activation differs from the substrate that is transported (58). Binding specificity can be determined *in vitro* by characterizing purified binding proteins by isothermal calorimetry, affinity gel electrophoresis (59), thermal shift assay in the presence of substrates to identify their impact on protein stability, pulldown analysis on beads covalently linked to the different sugars (60), or a scintillation proximity assay with radiolabeled substrates (61). However, protein purification can be challenging, especially for membrane-bound proteins (such as I9 PTS_EIIBCA). Moreover, in all the previous studies aiming to characterize binding specificity, the binding protein is dissociated from the multiproteic assembly, which could potentially bias the results of specificity determination.

**FIG 4:**
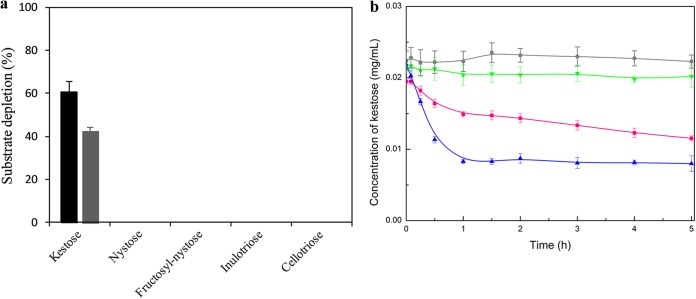
Oligosaccharide uptake and binding by I9min_PTS cells. (a) Screening of PTS specificity by testing the binding (gray bar) and uptake (black bar) of various gluco- and fructooligosaccharides by I9min_PTS cells. Oligosaccharide concentration was determined by HPAEC-PAD after 1 h of incubation of the oligosaccharides with I9min_PTS crude membrane cell extracts (for binding) or intact cells (for uptake). (b) Kestose binding and uptake kinetics by I9min_PTS crude membrane cell extracts and intact cells, respectively. Kestose concentration in the soluble phase was determined by HPAEC-PAD; EPI cells in green, EPI membrane extracts in gray, I9min_PTS cells in blue, and I9min_PTS membrane extracts in pink. The data represent the means from three biological replicates.

Here, we developed an easy-to-implement method based on the use of crude extracts of the cells harboring the target transporter and nonlabeled natural substrates. The unbound amounts of these are quantified by HPAEC coupled to PAD, a highly sensitive detection method. This generic approach can be used to quantitatively analyze the specificity of any transporter. We established the proof of concept by characterizing the *Dorea* PTS produced in E. coli. A truncated variant of I9, consisting only of the PTS (named I9min_PTS), was constructed in order to avoid any bias due to oligosaccharide hydrolysis in oligosaccharide uptake rate quantification. First, the uptake assays were performed with intact I9min_PTS cells incubated with various oligosaccharides in order to determine the PTS ability to internalize substrate (Fig. 4a). Using HPAEC-PAD, the amount of residual substrate was measured in the supernatant at different time points. We observed that among the different gluco- and fructooligosaccharides tested (kestose, nystose, fructosyl-nystose, inulotriose, and cellotriose), only kestose decreased in amount after incubation with I9min_PTS, confirming that the *Dorea* FOS PTS was specific to kestose. However, by incubating intact cells with the substrate, we could not determine the contribution of binding in the uptake process.

The ability of membrane debris to bind substrate was thus determined by incubating I9min_PTS lysed cells with oligosaccharides. To focus only on the binding step, cell debris was washed several times before the incubation to eliminate E. coli cytoplasmic phosphate, HPr, and EI in the reaction mixture, as this could lead to substrate phosphorylation and bias in binding rate quantification. As with the uptake assays, only kestose was bound by the I9min_PTS membranes (Fig. 4a). The PTS_EIIBCA was more slowly saturated by kestose with membrane extracts than with intact cells (Fig. 4b), indicating that kestose internalization shifts the binding equilibrium. Such a low rate of translocation had already been observed with the diacetylchitobiose-specific PTS from Vibrio furnissii 7225, where the transport kinetic was characterized by incubating growing V. furnissii cells on radiolabeled diacetylchitobiose (62). The low rate of this internalization process might be due to the covalent modification of substrate, in contrast to what happens with ABC transporters which hydrolyze ATP (54) or with secondary transporters such as the MFS, which use a chemiosmotic ion gradient to accelerate uptake (63).

**FIG 5:**
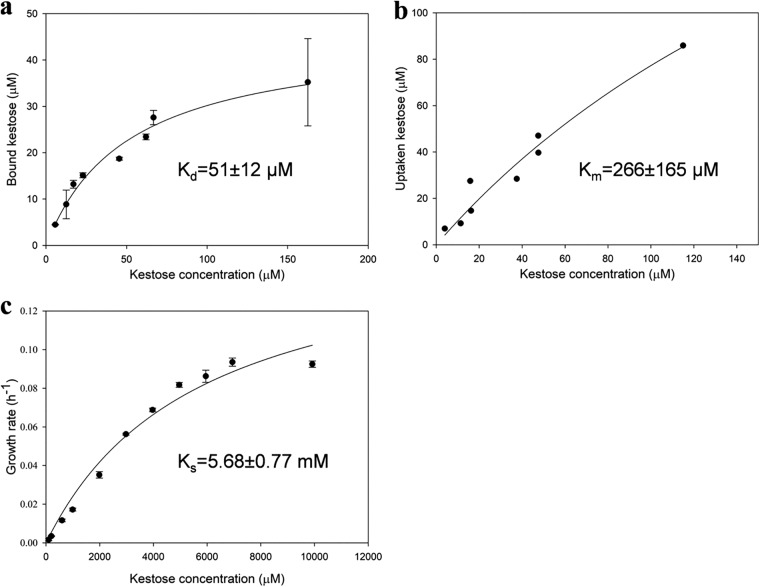
Saturation experiments on the *Dorea* PTS. Binding saturation of PTS_EIIBCA in I9min_PTS membranes (a), uptake saturation of I9min_PTS cells (b), and growth saturation of I9min_PTS/GH32 (c) with increasing concentrations of kestose. The data represent the means from two biological replicates.

Carbohydrate binding to the transporter is the first and essential step in an active transport system. The ligand-protein binding kinetic is fundamentally related to transport efficiency and microbial chemotaxis in ecological niches (64, 65). Here, we determined reliable kinetic parameters for binding, transport, and growth of I9 PTS to kestose (Fig. 5). Saturation experiments were performed on I9min_PTS cells and their membranes with increasing concentrations of kestose. The affinity constants of kestose uptake by I9min_PTS cells (*K_m_*) and of kestose binding by PTS_EIIBCA membranes (dissociation constant [*K_d_*]) were 266 *±* 165 and 51 *±* 12 μM, respectively. Even if these values are of the same order of magnitude, the fact that the *K_m_* value is higher that the *K_d_* one indicates that uptake is slightly less efficient than binding. It might be due to the complexity of the translocation process, which involves three PTS subunits (EIIC, EIIA, and EIIB), against only one (the EIIC part that faces the exterior of the cell) for binding. In addition, to infer the dependence of growth rate on kestose concentration, we estimated the substrate affinity constant (*K_s_*) by applying the Monod model (e.g., Michaelis-Menten hyperbolic dependence) to the growth rate of the I9min_PTS/GH32 cells versus kestose concentration data (66). The estimated *K_s_* value of 5.68 *±* 0.77 mM differs by 2 orders of magnitude from the uptake and binding constants. These kinetic binding parameters indicate that the I9min_PTS/GH32 strain has a strong binding affinity and efficient uptake system for kestose but a poor capacity to metabolize it. In the human gut, this kind of high-affinity uptake system is crucial for competing with other microbes for limited amounts of kestose. Furthermore, the inconsistency between the values of *K_s_*, *K_d_*, and *K_m_* shows that kinetic data for growth cannot be used to predict transporter binding and internalization ability.

### Functionality of the *Dorea* PTS transporter in E. coli.

**FIG 6:**
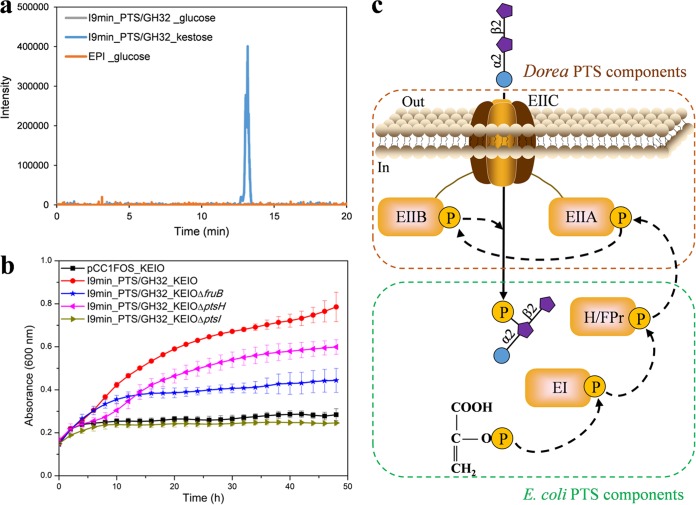
Functionality of the *Dorea* PTS in E. coli. (a) Extracted ion chromatogram (XIC) from negative ionization mode analysis at *m/z* 583.1270, corresponding to the exact mass of the [M-H]^−^ ion of phosphorylated kestose (calculated from the molecular formula C_18_H_33_O_19_P). Color code: I9min_PTS/GH32 clone grown on kestose, blue line; I9min_PTS/GH32 grown on glucose, gray line; EPI grown on glucose, orange line. (b) Growth curves on FOS of E. coli K-12 BW25113 (KEIO) single-knockout mutants for PTS genes (*ptsI*, *ptsH*, and *fruB*) transformed with fosmid I9min_PTS/GH32. The KEIO wild-type strain transformed with the empty fosmid pCC1FOS was used as the negative control. HPr (histidine protein) encoded by *ptsH* is a cytoplasmic protein component of the E. coli glucose PTS. It accepts a phosphate group from EI (encoded by *pstI*), a cytoplasmic protein required for E. coli PTS functioning, and transfers it to the carbohydrate-specific EIIA domain. *fruB* encodes the HPr-like component FPr of the E. coli fructose PTS. These data represent the means from three biological replicates. (c) Schematic representation of 1-kestose transport and phosphorylation by the *Dorea* PTS complemented with the E. coli HPr, FPr, and EI component. As shown in panel a, kestose can be phosphorylated at different carbon positions, while only one form of phosphorylated kestose is represented here.

PTS transporters couple transmembrane transport of substrate to its concomitant phosphorylation. However, the ability of PTS to phosphorylate a trisaccharide has never been shown until now. To demonstrate the full functionality of *Dorea* PTS_EIIBCA in E. coli and to identify the potential transient presence of intracellular phosphorylated kestose, we analyzed the intracellular metabolites of clone I9min_PTS/GH32 grown on kestose by ion chromatography coupled with electrospray ion ionization high-resolution mass spectrometry (IC-ESI-HRMS) (67) after quenching the metabolism (Fig. 6a).

As the E. coli without I9 PTS_EIIBCA did not grow on kestose, it could not be used as negative control. We thus compared the results obtained on kestose with those obtained with clones I9min_PTS/GH32 and of the KEIO collection (68) transformed with the empty fosmid pCC1FOS grown on glucose. For all strains, we searched for an *m/z* value of 583.1270, which corresponds to the theoretically expected value in negative mode for the phosphorylated kestose. For the I9min_PTS/GH32 and EPI strains grown on glucose, the e*x*tracted *i*on *c*hromatogram (XIC) does not show any peak. In contrast, we detected a chromatographically unresolved peak at retention time of 13.17 min for the I9min_PTS/GH32 strain grown on kestose (Fig. 6a), indicating the presence of several different forms of phosphorylated kestose.

Based on the above-described results, the I9 PTS_EIIBCA harboring the three well known domains of PTS transporters (A, B, and C domains) appears to be responsible for kestose internalization and phosphorylation by E. coli. As explained above, phosphorylation requires a multiproteic machinery to transfer the phosphoryl group from intracellular PEP to the carbohydrate bound to the EIIC domain of the PTS (41). Because the I9 locus lacks the EI and HPr components of the PTS (while being present elsewhere in the genome of Dorea longicatena DSM 13814, with the EI and HPr conserved histidine residues required for phosphate transfer), we hypothesized that the E. coli PTS machinery could complement I9 PTS_EIIBCA to achieve kestose phosphorylation. To investigate this question, we verified the ability of E. coli K-12 BW25113 (KEIO collection strains) single-knockout mutants for glucose and fructose-specific PTS genes transformed with fosmid I9min_PTS/GH32 to grow on a mixture of FOS, from which only kestose is utilized, as demonstrated above in the present paper. The *ptsI* (encoding EI) defective strain was unable to utilize FOS as carbon sources (Fig. 6b), demonstrating that this E. coli enzyme is required to ensure FOS-PTS functionality. Indeed, EI is involved in the first step of phosphorylation from PEP, which explains why deletion of *ptsI* in the KEIO strain completely abolished its growth on glucose and fructose (see Fig. S4). In contrast, mutants defective for *ptsH* (encoding histidine protein HPr, which transfers the phosphate group from EI to the EIIA component of the glucose PTS) or *fruB* (encoding HPr-like FPr in the fructose PTS) were able to grow on FOS, even though their growth ability was reduced compared to that of the wild-type strain transformed with I9min_PTS/GH32 (Fig. 6b). Similarly, deletion of *ptsH* or *fruB* in the KEIO strain resulted in a delayed lag phase when grown on glucose or fructose, respectively (Fig. S4), indicating that HPr could compensate for FPr deficiency and vice versa. We thus conclude that *Dorea* PTS_EIIBCA requires the E. coli EI to transfer phosphate from PEP to E. coli HPr (which can also be replaced, less effectively, by FPr), which, in turn, phosphorylates the I9 EIIA domain. Phosphate is then transferred to the I9 EIIB domain and, ultimately, to the kestose molecules internalized through the I9 EIIC component (Fig. 6c).

10.1128/mSphere.00771-19.4 FIG S4 Growth curves of knockout mutants (*ptsI*, *ptsH*, and *fruB*) of E. coli K-12 BW25113 (KEIO strain) on glucose (a) and fructose (b). The data represent the means from two biological replicates. Download FIG S4, PDF file, 0.1 MB. Copyright © 2020 Wang et al. 2020 Wang et al. This content is distributed under the terms of the Creative Commons Attribution 4.0 International license.

### PTS structural, functional, and taxonomical diversity.

By analyzing the specificity of *Dorea* PTS_EIIBCA, we extended the panel of characterized glycoside transporters, which is still limited. Prior to this study, only two PTSs (PTS1 and PTS26 from *L. plantarum* ST-III) were known to internalize FOS (37). However, these proteins are not listed in the Transporter Classification database (TCDB) (69). In the TCDB, based on functional and phylogenetic information about the EIIC domain (as in the first classification of PTS proposed by Nguyen et al. [70]), PTSs have been classified into seven families of phosphotransfer-driven group translocators, consisting of the glucose-glucoside (4.A.1, Glc), fructose-mannitol (4.A.2, Fru), lactose-*N*,*N*-diacetylchitobiose (4.A.3, Lac), glucitol (4.A.4, Gut), galactitol (4.A.5, Gat), mannose-fructose-sorbose (4.A.6, Man), and l-ascorbate (4.A.7, l-Asc) families.

**FIG 7:**
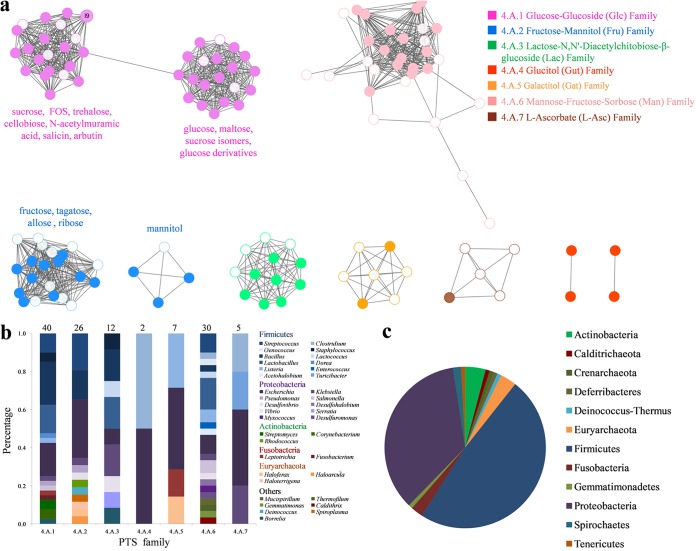
Functional, structural, and taxonomical diversity of PTS. (a) Sequence similarity network (SSN) of PTS-EIIC domains from 125 proteins, retrieved from the Transporter Classification database, from the literature, and from the present study, constructed with an initial score of 10^−5^. The nodes are connected by an edge if the pairwise sequence identity is ≥21.3%. The corresponding substrates were annotated under each cluster of the 4.A.1 and 4.A.2 families. Unfilled nodes indicate sequences for which the functions were inferred by transcriptomics, while filled ones correspond to the functions validated biochemically or by gene deletion. Taxonomic distributions at genus (b) and phylum (c) levels of the characterized PTS transporters.

To locate the FOS transporters in the diversity of PTSs and to investigate the sequence-function relationships of these proteins, we constructed a sequence similarity network (SSN) from the 125 sequences of EIIC components retrieved from TCDB (120 sequences for 117 PTSs, since 3 have 2 EIIC domains), the literature (4 sequences), and the present study (Fig. 7a). In an SSN network, nodes (representing protein sequences) in the same cluster are connected with an edge if they share a percentage of identity higher than a threshold value, providing a more visually tractable view of divergent proteins than alignment-based dendrograms or phylogenetic trees (44). First, despite the low sequence identity threshold used to link the nodes in the sequence network to form clusters (21%), we observed that the 3 FOS PTSs (one from *Dorea* and two others from *L. plantarum* DT-III) do not belong to the cluster of sequences containing the members of the Fru family 4.A.2. Even though FOSs contain more fructosyl than glucosyl moieties (one terminal glucosyl moiety in a FOS molecule, regardless of its degree of polymerization), the FOS PTSs belong to the Glc family 4.A.1. This could correlate to the fact that the *Dorea* PTS requires the terminal glucosyl moiety of FOS to bind and internalize its substrate.

Second, we observed that the two large Glc and Fru PTS families are subdivided into two clusters each in the SSN. The 4.A.1 Glc family contains two subfamilies in the TCDB, each corresponding to one of the SSN clusters: (i) subfamily 4.A.1.1, named “glucose subfamily” in the TCDB, contains PTSs specific to glucose, its derivatives (such as *N*-acetylglucosamine), and also α-glucosides such as maltose and sucrose isomers; (ii) subfamily 4.A.1.2, named “glucoside subfamily,” contains PTSs targeting α- and β-glucosides, such as sucrose (GF), trehalose (α-1,1-linked GG, which has a close structural similarity to sucrose [71]), FOS (GFn), cellobiose (β-1,4-linked GG), and other β-glucosides (salicin and arbutin), and *N*-acetylmuramic acid. If the present SSN clusters correspond to the TCDB 4.A.1 subfamilies, they are not the same as those previously proposed by Nguyen et al., who categorized the proteins now listed in the 4.A.1 family into the glucose (Glc) and β-glucoside (Bgl) families (70). Regarding the 4.A.2 Fru family, for which no subfamily is described in the TCDB, one SSN cluster contains the transporters targeting fructose, tagatose, ribose and allose, while the other one, clearly separated, contains mannitol-PTS. In this case, this is consistent with the findings of Nguyen et al., who described the mannitol family apart from Fru (70).

From these results, we conclude that there is no longer a link between the names of the families listed in the available PTS classifications and the function of their constituent members. Improvements in transporter classification will be proposed in the following section.

Since phylogenetic links are one of the bases of the existing transporter classifications, we also analyzed the taxonomical diversity of the 125 known PTS_EIIC sequences. They represent 37 genera, classified into 12 distinct phyla (Fig. 7b and c). For each PTS family, the taxonomic diversity is wide ranging, with no family assigned to a specific phylum or genus. Of 125 sequences, more than half were assigned to the four genera *Escherichia* (20%), *Streptococcus* (10%), *Bacillus* (13%), and *Lactobacillus* (11%). Consequently, more than 80% belong to only two phyla, *Firmicutes* (48%) and *Proteobacteria* (35%). There is a similar distribution of characterized PTSs between Gram-negative and Gram-positive bacteria. The present taxonomic distribution of PTS is highly biased by sequencing and biochemical characterization efforts which are unequal in their coverage of actual bacterial diversity. For example, characterized PTSs are absent from the *Bacteroidetes* phylum, while in the Pfam 32.0 database, we found 10 *Bacteroidetes* sequences harboring a PTS_EIIC Pfam motif (PF13303) (72). In addition, the characterized PTSs appear to be very abundant in *Firmicutes* (48%) (Fig. 7c), while this phylum accounts for just 29% of the sequenced bacterial genomes (versus 54% for *Proteobacteria*) referenced in the Genomes Online database (GOLD) (73). In any event, though, there are still many transporters to be discovered in the near future thanks to powerful tools such as functional metagenomics and novel generic biochemical approaches, such as those described here.

## DISCUSSION

### Biochemical characterization of a FOS-specific PTS transporter.

The PTS is a multiple component carbohydrate transport and phosphorylation system composed of two cytoplasmic phosphotransferases, enzyme I (EI) and histidine-containing phosphocarrier protein (HPr), which are common in most systems, and one multiproteic enzyme II complex (EII) including two phosphotransferase proteins (EIIA and EIIB) and one integral membrane protein (EIIC), which is usually specific for one substrate or a small group of closely related carbohydrates. For example, 21 different EII complexes were identified in E. coli as being involved in the transport of approximately 20 different carbohydrates, with only 1 EI and 1 HPr protein, and five EI and six HPr paralogues, whose functions are still to be uncovered (43). PTSs are thus major carbohydrate transport systems in certain bacteria, but their diversity and functionalities have been insufficiently investigated. Today, we have just 122 PTSs characterized (either biochemically or by transcriptomics and/or gene deletion), targeting a substrate range (mainly monosaccharides and some glucosides, *N*-acetyl-glucosides, fructosides, and galactosides) that is highly restricted compared to the possible diversity of oligosaccharides (74).

In the present study, we characterized a PTS_EIIBCA complex involved in FOS utilization by both uncultured and cultured *Dorea* strains. Previously, several FOS PTSs have been identified from lactic acid bacteria and characterized by growth tests and transcriptomic analysis (75, 76), sometimes also with knockout experiments (37), or by measurement of the extracellular composition by anionic exchange chromatography (45). However, the precise molecular mechanism and the exact lengths of the oligosaccharides that could be bound and internalized were not deeply investigated due to the technical bottlenecks inherent in transporter characterization. Here, we developed a highly generic, sensitive, and quantitative method based on HPAEC-PAD analysis of oligosaccharides unbound to crude membranes or to entire cells (native or recombinant). This approach, which requires no protein complex dissociation or purification steps, is compatible with the use of unlabeled natural substrates to quantify binding, transport, and metabolization rates. It thus effectively replaces the use of radiolabeled substrates, which have been widely used until now for transporter characterization. Here, we were able to determine the contributions of substrate binding and transport to the oligosaccharide metabolization process. We proved that *Dorea* PTS_EIIBCA is able to transport sucrose and especially kestose, with an affinity for kestose binding five times better than its affinity for uptake.

Moreover, carbohydrate uptake through a PTS implies a phosphorylation cascade. PTS-based phosphorylation of oligosaccharides with a DP of more than 2 had never been shown. Here, we proved that a DP3 oligosaccharide can be phosphorylated by *Dorea* PTS_EIIBCA and that its specificity for phosphorylation is the same as that for binding and translocation. We demonstrated this directly *in cellulo*, in contrast to the previous studies, in which phosphorylation reactions were performed *in vitro* by adding purified phosphotransferases and EIIAB to the EIIC component (77). By IC-ESI-HRMS analysis and use of E. coli single-knockout mutants, we proved the entire functionality of the *Dorea* PTS_EIIBCA complex in E. coli, thanks to efficient complementation by the host’s EI and HPr (or, alternatively, FPr) phosphotransferases.

Several PTS_EII complexes involved in cellobiose utilization by Gram-positive bacteria were previously successfully expressed in an E. coli host strain (78, 79) and proven *in vitro* to be complemented by the E. coli EI and HPr PTS components (80). The difference in cell membrane organization between Gram positives and E. coli thus appears not to have an impact on the functionality of recombinant PTS_EIIBCA complexes, even though the localization of each component of Gram-positive PTS in E. coli is still an open question.

### Revisiting the PTS classification.

In the last few years, a huge mass of omics data has accumulated (81), and microfluidic approaches have been developed to rapidly identify the substrates targeted by (meta)genomic loci (82). Meanwhile, an increasing number of tridimensional transporter structures have been solved (83), and the panel of biochemical methods for transporter characterization has been extended, as described in the present paper. It is thus likely that significant progress will be made over the next few years in the understanding of transporter structure-function relationships. In the present paper, we investigated this question by analyzing the diversity of sequences and substrate specificities of known PTSs. We showed that as the number of transporters with characterized specificities increases, there is no longer a link between the names of PTS families and their functions (for example, subfamily 4.A.1.1, named “glucose subfamily” in the TCDB, contains PTSs specific to *N*-acetylglucosamine and to some glucosides, while the subfamily 4.A.1.2, named “glucoside subfamily,” contains PTSs specific to *N*-acetylmuramic acid). The present classification is thus quite confusing. Instead, we propose a transporter classification based on similarities of structures and mechanisms, with families containing members with one or more substrate specificities. This kind of classification, with no specificity-related constraints, does exist for enzymes. For example, the CAZy database, based on this principle, is a reference for carbohydrate active enzyme classification (84), even though it is complementary to the EC classification (the EC numbers now appear in the CAZy database for each biochemically characterized enzyme). For transporter classification, we recommend the continued use of the TCDB, which is a mine of information for these proteins, but with caution with respect to the level of classification. Indeed, of the five components of the transporter classification identifier (TCID), only the first two relate to structure and mechanism: the first number corresponds to the transporter class (i.e., channel, carrier [porter], primary active transporter, or group translocator), and the following letter corresponds to the transporter subclass, which in the case of primary active transporters, refers to the energy source used to drive transport. The last three TCID components are, in most cases, related to substrates (at least for PTSs) in the present TCDB. Classification into superfamilies, families, and subfamilies will thus require further global/local sequence analyses in order to identify specific common structural traits within transporter groups. Of course, listing the known substrates for each functionally characterized transporter is still highly valuable, as it will provide a clear view of the functional diversity in each of the structurally defined groups. Thus, major advances can be made to elucidate, and ultimately predict, the structural determinants of transporter specificity.

### Significance of unlocking prebiotic metabolic pathways from the human gut microbiome and implications for gut health.

FOS, one of the most commonly used and studied prebiotics, has been shown to stimulate the growth of beneficial gut bacteria such as bifidobacteria and lactic bacteria (30) but also some pathogenic E. coli strains (13). Previously, most of the FOS metabolization pathways were discovered from cultured bacteria, but the emergence of high-throughput functional metagenomic approaches has made it possible to study more extensive sequence spaces and highlighted new genomic loci involved in FOS metabolism by uncultured bacteria (20). Here, we studied a locus assigned to Dorea longicatena DSM 13814 (99% identity). However, we could not exclude that it might come from a different bacterium, which would have acquired this DNA fragment by horizontal gene transfer.

Previously, *D. longicatena* was found to be stimulated by short-chain FOS, with a poor ability to use long-chain β-fructans (14, 19). This is consistent with the specificity of the *Dorea* locus characterized here for kestose, a DP3 FOS, which is by far the most abundant FOS in wheat (85), a staple food for Westerners and a major component of the FOS mixtures used for dietary supplementation (86). It does not exclude the possibility, however, that other *D. longicatena* loci could be involved in FOS metabolization, since we found three other loci in strain DSM13814 harboring an I9_GH32 homolog (33% to 42% identity) with either an ABC transporter or a PTS. *Dorea* has been shown in several studies to be positively associated with intestinal permeability in individuals with alcohol dependence, with multiple episodes of sclerosis relapse (87, 88), and with IBS (15, 16). In contrast, in several studies, it was negatively associated with IBD. This dysbiosis, which is even more pronounced in a common complication in IBD, Clostridium difficile infection, is likely to be due to administration of antibiotics (89, 90). At the species level, *D. longicatena* is also negatively associated with Crohn’s disease (91) and positively with remission after surgery (92). Here, however, we showed that the *Dorea* FOS utilization locus is twice as abundant in the microbiomes of patients suffering from IBD than in those of healthy individuals. It is thus difficult to correlate this to the results of genera and species abundance in the context of IBD, especially since the present study and previous ones showed that Gram-positive and -negative PULs are frequently exchanged between gut bacteria (20, 27), providing the microbiota with metabolic flexibility and robustness. Taking that into account, it appears more informative to focus on pathology biomarkers of the microbiome and their specific functions rather than on bacterial species whose genomic composition and metabolic abilities are far from fully characterized and which are, in addition, variable.

The focus should now be on the function of the PTS characterized here and links to gut health. Based on the fact that prebiotics are characterized as stimulating the growth of beneficial gut bacteria, the effect of FOS dietary supplementation in decreasing Crohn’s disease activity has been tested (93). Nevertheless, the latest results from a randomized, double-blind placebo-controlled trial revealed no significant benefit of FOS supplementation in patients with active Crohn’s disease other than an induction of immunoregulatory dendritic cell responses (94). To date, only a small number of clinical trials have investigated prebiotic supplementation as treatment for chronic gastrointestinal disorders (including IBD and IBS). It remains a controversial issue with conflicting results depending on the type and dose of the prebiotic used (95). In any case, the biochemical characterization of prebiotic utilization machineries, such as the FOS-targeting transport system highlighted here both as a biomarker of diet and pathology, is of strategic importance for formulating the mechanistic bases for microbiome functional engineering, such as through the design of future specific probiotic-prebiotic combinations to maximize host benefits.

## MATERIALS AND METHODS

### Escherichia coli strains.

The metagenomic clone I9 includes contigs I9a (GenBank accession number HE717008.1) and I9b (GenBank accession number HE717009.1). This E. coli clone comes from a metagenomic library constructed by sampling the ileum mucosal microbiota of a 51-year-old male patient undergoing colonoscopy and surgery for suspected lower colon cancer (20). The metagenomic DNA fragments (30 to 40 kbp) were cloned into the pCC1FOS fosmid and transformed into the EPI100 E. coli strain (Epicentre Technologies). All the I9a variants were constructed using the In-Fusion HD Cloning Plus kit (Clontech) according to the instructions in the user manual. For each variant, amplification started 600 bp to 1 kb upstream of the first gene of the target locus to involve potential promoter sequences which might be necessary for gene expression (52). The primers used in this study are listed in Table S1 in the supplemental material. Mutants were confirmed by new-generation sequencing, performed by the GeT-Biopuces Platform (Toulouse) using the Ion Torrent S5 system. Read assembly was performed using Masurca (http://www.genome.umd.edu/masurca.html). The assembled contigs were cleaned from the pCC1FOS vector sequence using Crossmatch (http://bozeman.mbt.washington.edu/phredphrapconsed.html).

10.1128/mSphere.00771-19.5 TABLE S1 List of primers used in this study. Download Table S1, DOCX file, 0.01 MB. Copyright © 2020 Wang et al. 2020 Wang et al. This content is distributed under the terms of the Creative Commons Attribution 4.0 International license.

10.1128/mSphere.00771-19.6 TABLE S2 Ability of intracellular extracts of I9min_GH32 cells to hydrolyze various FOS and β-fructans. Download Table S2, DOCX file, 0.01 MB. Copyright © 2020 Wang et al. 2020 Wang et al. This content is distributed under the terms of the Creative Commons Attribution 4.0 International license.

### Growth conditions.

All the E. coli variants constructed in this study were grown in M9 medium (Na_2_HPO_4_·12H_2_O, 17.4 g/liter; KH_2_PO_4_, 3.03 g/liter; NaCl, 0.51 g/liter; NH_4_Cl, 2.04 g/liter; MgSO_4_, 0.49 g/liter; CaCl_2_, 4.38 mg/liter; Na_2_EDTA·2H_2_O, 15 mg/liter; ZnSO_4_·7H_2_O, 4.5 mg/liter; CoCl_2_·6H_2_O, 0.3 mg/liter; MnCl_2_·4H_2_O, 1 mg/liter; H_3_BO_3_, 1 mg/liter; Na_2_MoO_4_·2H_2_O, 0.4 mg/liter; FeSO_4_·7H_2_O, 3 mg/liter; CuSO_4_·5H_2_O, 0.3 mg/liter; thiamine, 0.1 g/liter; and leucine, 0.02 g/liter) supplemented with 12.5 mg/liter chloramphenicol. The carbohydrates were diluted in M9 medium at 0.5% (wt/vol). The cultures were inoculated at an initial optical density at 600 nm (OD_600_) of 0.05 from precultures in LB.

Growth dynamic were analyzed at 37°C on various oligosaccharides and polysaccharides: FOS (Beghin Meiji; containing 5.3% [wt/wt] sucrose, 40.0% [wt/wt] 1-kestose, 46.0% [wt/wt] nystose, and 8.7% [wt/wt] fructosyl-nystose, quantified by HPAEC-PAD analysis, as described further in this section), sucrose (Sigma), 1-kestose (GF_2_; Wako Chemicals), nystose (GF_3_; Wako Chemicals), fructofuranosyl-nystose (GF_4_; Wako Chemicals), inulotriose (Megazyme), inulin from dahlia tuber (Sigma), and levan from Erwinia herbicola (Sigma). The optical density of the cultures was measured at 600 nm using a plate reader (Infinite M200pro; TECAN).

To evaluate the role of endogenous E. coli PTS genes in the phosphorylation of kestose, E. coli K-12 BW25113 strain and its derived single-knockout mutants (from the KEIO library) were employed (68). I9min_PTS/GH32 fosmid was electrotransferred into E. coli K-12 BW25113 single-knockout mutants for the *ptsI*, *ptsH*, and *fruB* genes. Their growth abilities in M9 medium supplemented with FOS, glucose, or fructose were analyzed. The wild-type KEIO strains transformed with the I9min_PTS/GH32 fosmid and the empty fosmid pCC1FOS were used as positive and negative controls, respectively. The cultures were inoculated at an initial optical density at OD_600_ of 0.05 from precultures in LB, and cell growth was monitored by measuring the OD_600_ over 48 h at 37°C using the FLUOStar Optima (BMG Labtech).

### FOS utilization analysis.

To determine how the different oligosaccharides in the FOS mixture were utilized by E. coli, HPAEC-PAD was used to measure the concentration of residual carbohydrates in the culture medium filtered at 0.20 μm. Supernatants were analyzed on a Dionex ICS-3000 system using a CarboPac PA100 4 × 250 column. Carbohydrates were eluted at 30°C at a flow rate of 1 ml/min with the following multistep gradients: 0 to 30 min (0% to 60% B), 30 to 31 min (60% to 0% B), and 31 to 36 min (0% B). Solvents were 150 mM NaOH (eluent A) and 150 mM NaOH, 500 mM CH_3_COONa (eluent B).

### Enzymatic assays.

FOS hydrolysis assays were performed by using the cytoplasmic fraction of the I9min_GH32 variant and the extracellular fraction. The I9min_GH32 variant was grown overnight at 37°C in LB supplemented with chloramphenicol at 12.5 mg/liter. The crude cytoplasmic extracts were obtained as follows: after centrifugation, cells were concentrated to an optical density at 600 nm (OD_600_) of 80 in 50 mM potassium phosphate buffer (pH 7.0) containing 0.5 g/liter lysozyme and incubated at 37°C for 1 h. Cell lysis was completed with one freeze (at −80°C) and thaw (at 37°C) cycle. Cell debris was centrifuged at 15,000 × *g* for 30 min, and the cytoplasmic extracts were filtered with a 0.20-mm Minisart RC4 syringe filter. Enzymatic assays were performed at 37°C with either the cytoplasmic extracts or the culture supernatant and 0.5% (wt/vol) FOS, sucrose, 1-kestose, nystose, fructofuranosyl-nystose, inulotriose, inulin, or levan. Sampling was performed at the initial time point and at 24 h. Reactions were stopped by heating to 95°C for 5 min. Samples were analyzed by HPAEC-PAD as described above.

### Gene expression analysis.

Total RNAs were extracted using the RNeasy Minikit (Qiagen) from three independent cultures of the I9 clone in mid-exponential phase in LB. The RNase-free DNase set (Qiagen) was used to remove contaminating DNA. RNAs were quantified using a NanoDrop (Thermo Fisher Scientific), and the Bioanalyzer RNA kit (Agilent Technologies) was used to control their quality. Then, 5 μg of total RNA was retrotranscribed using SuperScript II reverse transcriptase (Thermo Fisher Scientific) and random primers (Invitrogen). Synthesized cDNA was purified using Illustra MicroSpin G-25 columns (GE Healthcare). The primers used for real-time quantitative PCR (qPCR) were designed using the Primer3Plus web interface with lengths from 18 to 22 bases, a GC content of more than 50%, melting temperatures between 55 and 65°C, and an amplification size from 83 to 148 bases (Table S1). Prior to RNA expression analysis, primer specificity was checked on the genomic DNA of the E. coli I9 clone. qPCR was carried out on a CFX96 Touch real-time PCR detection system (Bio-Rad) and monitored using the CFX Maestro software. Data were normalized based on the expression of the housekeeping gene encoding integration host factor β-subunit (*ihfB*), which is a commonly used reference gene in E. coli owing to its constant expression throughout growth (96). Relative expression was calculated as 2^−Δ^*^CT^*, in which Δ*C_T_* was obtained by subtracting the average cycle threshold value (*C_T_*; corresponding to the number of cycles required for the fluorescent signal to cross the threshold) of the housekeeping gene from that of the gene of interest (97). For each of the three biological replicates, four technical replicates were performed, corresponding to four different DNA dilutions.

### Carbohydrate uptake and binding rate quantification.

Overnight cultures of the I9min_PTS variant in LB medium supplemented with 12.5 mg/liter chloramphenicol were centrifuged. Cells were kept at 4°C no longer than 4 h before use. For uptake experiments, cell pellets were washed three times in 50 mM Tris-buffered saline (TBS; pH 7.0) and then resuspended in TBS to reach an OD_600_ of 40. Sucrose, fructose, glucose, kestose, nystose, fructofuranosyl-nystose, or inulotriose was added at a final concentration ranging from 0.5 to 100 mM. All reaction mixtures were incubated at 37°C with shaking at 200 rpm. After different incubation times, from 5 min to 5 h, samples of 0.2 ml were filtered with a 0.20-mm Minisart RC4 syringe filter. The concentrations of unbound carbohydrates in the supernatant were determined by HPAEC-PAD as described above. For binding experiments, cell pellets were resuspended in TBS containing 0.5 g/liter lysozyme and incubated at 37°C for 1 h. Cell lysis was completed with one freeze (at −80°C) and thaw (at 37°C) cycle. Cell membrane fractions were isolated by centrifugation at 15,000 × *g* for 30 min, washed three times with 50 mM Tris buffer (pH 7.0), and resuspended in the same buffer to reach an OD_600_ of 40. The later steps were the same as for the uptake experiments. The EPI clone was used as negative control in all these experiments.

### Kestose phosphorylation analysis.

One hundred microliters of an overnight culture of a knockout E. coli strain in LB was used to inoculate 50-ml baffled shake flasks containing 10 ml of M9 medium supplemented with glucose or kestose as carbon sources. The flasks were incubated for 24 h at 37°C with orbital shaking at 220 rpm. Cells were harvested by centrifugation for 10 min at 2,000 × *g* at room temperature, washed with diluted M9, and used to inoculate 50-ml baffled shake flasks (at an OD_600_ of 0.1) containing M9 supplemented with the same carbon source. Cell growth, performed at 37°C and 220 rpm, was monitored by measuring OD_600_ with a cell density meter (Ultrospect 10; Amersham BioSciences). When the culture reached the exponential phase, 120 μl of culture medium was withdrawn and vigorously mixed with 1.25 ml of an acetonitrile/methanol/H_2_O (4:4:2) solution precooled at −20°C to rapidly quench metabolic activity and extract metabolites (67). The samples were kept at −20°C for 20 min and then centrifuged at 10,000 × *g* at 4°C for 10 min to remove cell debris. The supernatant was dried using a SpeedVac (SC110A SpeedVac Plus; ThermoSavant) under vacuum and then stored at −80°C until analysis.

The samples were dissolved in 120 μl of ultrapure water and then analyzed by ion chromatography (Thermo Scientific Dionex ICS-5000+ system; Dionex) coupled to an LTQ Orbitrap mass spectrometer (Thermo Fisher Scientific) equipped with an electrospray ionization probe (IC-ESI-HRMS). A revised ion chromatography method was used here (98). The KOH gradient was modified as follows: 0 min, 0.5 mM; 1 min, 0.5 mM; 9.5 min, 4.1 mM; 12.5 min, 30 mM; 24 min, 50 mM; 36 min, 60 mM; 36.1 min, 90 mM; 43 min, 90 mM; 43.5 min, 0.5 mM; 48 min, 0.5 mM. The gradients were all linear. For background suppression, an anionic electrolytically regenerated suppressor (AERS 300-2 mm; Dionex) was used. The device was operated at a constant 87-mA electrolysis current in external water mode with ultrapure water and regenerant delivered by an external AXP pump at a flow rate of 1 ml/min. The volume of the injected sample was 35 μl. Mass spectrometry analysis was performed in the negative Fourier transform mass spectrometry (FTMS) mode at a resolution of 30,000 in full scan mode with the following source parameters: capillary temperature, 350°C; source heater temperature, 300°C; sheath gas flow rate, 50 arbitrary units (AU); auxiliary gas flow rate, 5 AU; S-Lens radio frequency (RF) level, 60%; and ion spray voltage, 3.5 kV. The data were acquired using Xcalibur software (Thermo Fisher Scientific).

### Bioinformatics analysis.

The LipoP 1.0 server was used to predict lipoprotein and signal peptides (http://www.cbs.dtu.dk/services/LipoP/) (99). Transmembrane domains were predicted using the TMHMM server v.2.0 (http://www.cbs.dtu.dk/services/TMHMM/) (100). Protein function was predicted by analyzing conserved domains in the NCBI’s conserved domain database (101). Sequence similarity networks (SSNs) were generated via the Enzyme Similarity Tool (EFI-EST; https://efi.igb.illinois.edu/efi-est/) (102) by inputting 125 amino-acid sequences of EIIC proteins extracted from the Transporter Classification database (TCDB; http://www.tcdb.org/) (103), the literature (37, 45, 104), and the present study. The SSNs were generated with edge values (E values) of 10^−3^ to 10^−20^, and the alignment scores were further refined after the sequence networks were visualized in Cytoscape 3.6.1 (105). An E value threshold of 10^−5^ corresponding to 21.3% sequence identity was finally used to visualize the network.

Sequences of the I9a open reading frames (ORFs) were searched by BLASTP analysis (E value = 0, identity ≥ 90%, no coverage threshold) against the translated catalogue of 9.9 million reference genes constructed from the fecal metagenome of 1,267 subjects from the United States, China, and Europe (46, 106). The microbial gene richness in the human gut microbiome was determined using the abundance and frequency matrix in the 1,267 subjects (http://meta.genomics.cn/meta/dataTools).

### Data availability.

The nucleotide sequences of the I9 metagenomic DNA are available in the GenBank database under accession numbers HE717008.1 for contig I9a and HE717009.1 for contig I9b.
