# Supplementary figures and images for: Harvesting of Prebiotic Fructooligosaccharides by Nonbeneficial Human Gut Bacteria
Source: mSphere. 2020 Jan 8;5(1):e00771-19. doi: 10.1128/mSphere.00771-19 (PMC6952197; doi:10.1128/mSphere.00771-19)

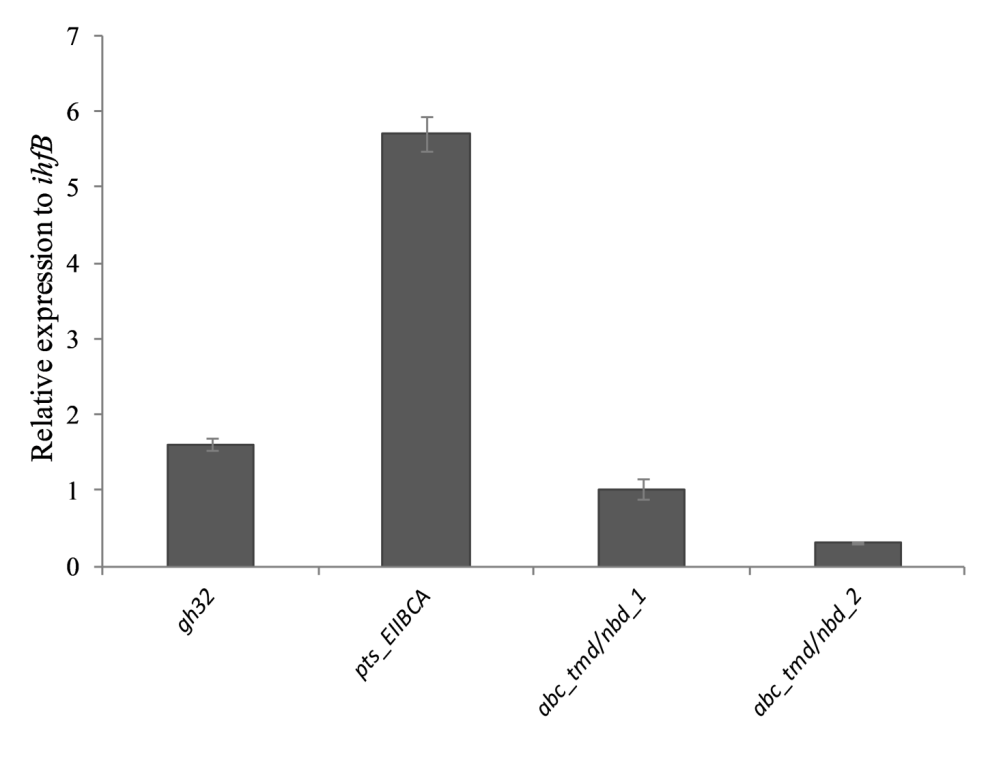

Supplement: FIG S1 [file mSphere.00771-19-sf001.tif]

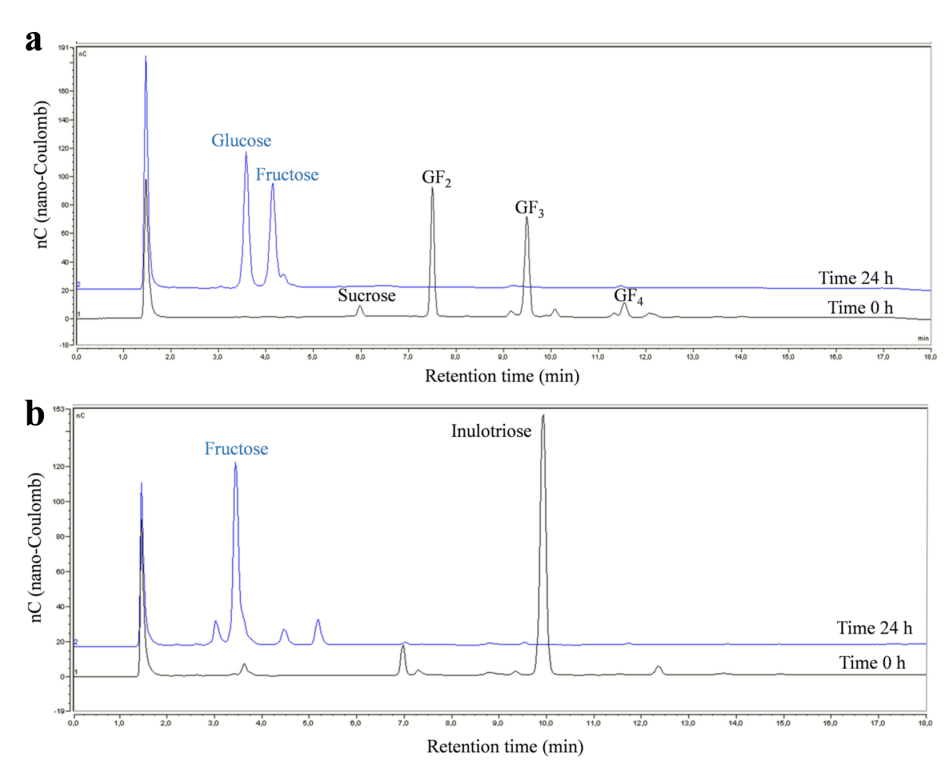

Supplement: FIG S2 [file mSphere.00771-19-sf002.tif]

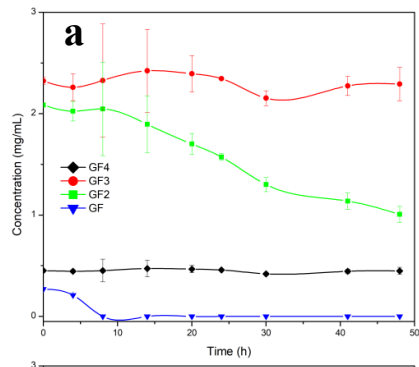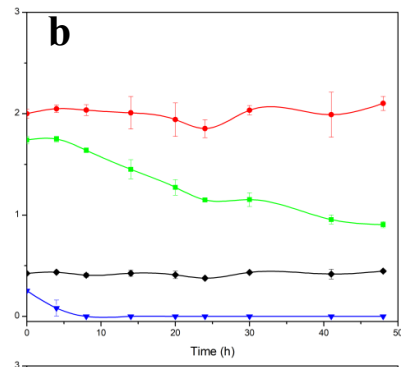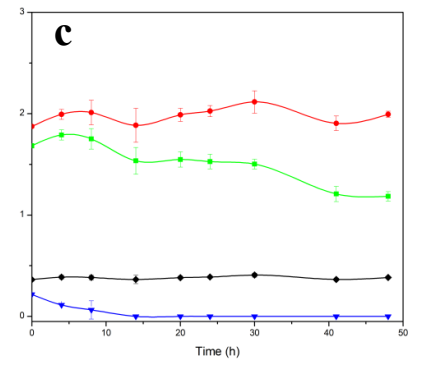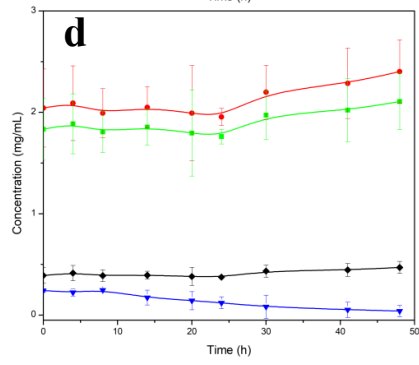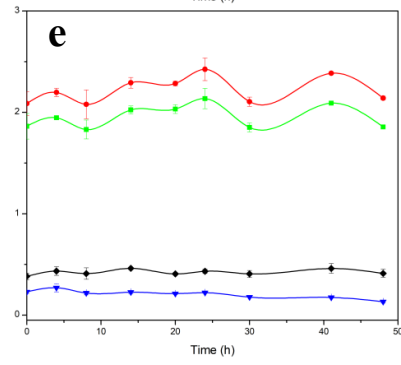

Supplement: FIG S3 [file mSphere.00771-19-sf003.pdf]

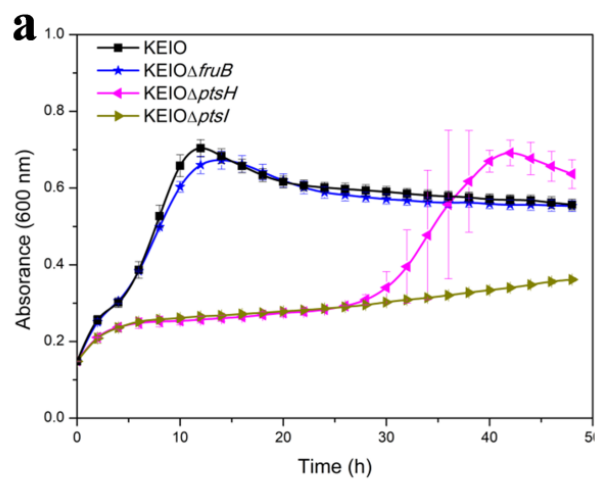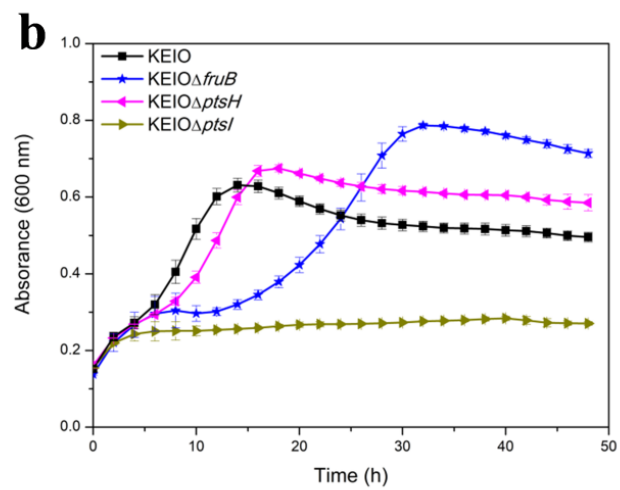

Supplement: FIG S4 [file mSphere.00771-19-sf004.pdf]
