# Supplementary material for: Harvesting of Prebiotic Fructooligosaccharides by Nonbeneficial Human Gut Bacteria
Source: mSphere. 2020 Jan 8;5(1):e00771-19. doi: 10.1128/mSphere.00771-19 (PMC6952197; doi:10.1128/mSphere.00771-19)
Supplement: TABLE S1 [file mSphere.00771-19-st001.docx]

**Table S1.** List of primers used in this study.

| **Name** | **Sequence 5’→3’** |
| --- | --- |
| *I9 variant construction* |  |
| Pcc1fos_f | GTGGGATCCTCTAGAGTCGACC |
| Pcc1fos_r | GTGGGATCCCCGGGTACCGAGC |
| I9min_F | ACCCGGGGATCCCACGCATCACAGGCGGACTTGCCAGAG |
| I9min_R | TCTAGAGGATCCCACACCTCTCGGATCGTCGCAGACACA |
| I9min_PTS/GH32_R | TCTAGAGGATCCCACCAGAATCCATCAGCCGGATCACGG |
| I9min_ABC/GH32_F | GTGCATATAAACCTCCCATGGAATTAATCTTTGGTACAACCACCCCGATC |
| I9min_ABC/GH32_R | CATGGGAGGTTTATATGCACCAACCGTAG |
| I9min_GH32_R | TCTAGAGGATCCCACCATGGGAGGTTTATATGCACCAACCGTAG |
| I9min_PTS_F | ACCCGGGGATCCCACACCGCCTTCTGCATTAAACGGCG |
| *Gene expression analysis* |  |
| I9a_*gh32*_f | ATCCCTCTCCTGCCAGCATA |
| I9a_*gh32*_r | ACGGCAAGATCTGTCAACGT |
| I9a_*pts_EIIBCA*_f | TACGATGGCCGGAATGATCG |
| I9a_ *pts_EIIBCA* _r | TCGACATCGCAGGTATCACG |
| I9a_*abc_tmd/nbd_1*_f | GCATGTCTGGATACCTCCCG |
| I9a_ *abc_tmd/nbd_1*_r | CAGGATGCGGTAAGACGACA |
| I9a_ *abc_tmd/nbd_2*_f | TCAGTGTGTCTGTTCCGAGC |
| I9a_ *abc_tmd/nbd_2*_r | GACCAGTGTCAGACAGGCAA |
| *ihfB*_f | GCCAAGACGGTTGAAGATGC |
| *ihfB*_r | CAAAGAGAAACTGCCGAAACC |
