# Supplementary material for: Harvesting of Prebiotic Fructooligosaccharides by Nonbeneficial Human Gut Bacteria
Source: mSphere. 2020 Jan 8;5(1):e00771-19. doi: 10.1128/mSphere.00771-19 (PMC6952197; doi:10.1128/mSphere.00771-19)
Supplement: TABLE S2 [file mSphere.00771-19-st002.docx]

**Table S2.** Ability of intracellular extracts of I9min_GH32 cells to hydrolyze various FOS and β-fructans.

| Substrate | Initial concentration | | Substrate consumption after 24 h (%) |
| --- | --- | --- | --- |
|  | mg/ml | mM |  |
| Sucrose | 5 | 14.6 | 99.4 |
| Kestose | 5 | 9.9 | 100 |
| Nystose | 5 | 7.5 | 100 |
| Fructosyl-nystose | 5 | 6.0 | 92.2 |
| Inulotriose | 5 | 9.9 | 100 |
| Inulin | 5 | - | 0 |
| Levan | 5 | - | 0 |
